# Gut microbiota contributes to the therapeutic effect of acupuncture in atopic dermatitis

**DOI:** 10.1128/spectrum.03912-25

**Published:** 2026-08-03

**Authors:** Yerin Seo, Jundong Kim, Mijung Yeom, Su-Yang Park, Sujin Lee, Sora Ahn, Dae-Hyun Hahm, Kyuseok Kim, Soon-Kyeong Kwon, Hi-Joon Park

**Affiliations:** 1 Division of Applied Life Science (BK21), Gyeongsang National University 26720, Jinju, Republic of Korea; 2 Department of Ophthalmology, Otorhinolaryngology and Dermatology of Korean Medicine, Graduate School of Korean Medicine, Kyung Hee University 26723, Dongdaemun District, Republic of Korea; 3 Department of Ophthalmology, Otorhinolaryngology and Dermatology of Korean Medicine, Kyung Hee University Medical Center, Seoul, Republic of Korea; 4 Acupuncture and Meridian Science Research Center, College of Korean Medicine, Kyung Hee University, Seoul, Republic of Korea; 5 Department of Physiology, School of Medicine, Kyung Hee University 26723, Seoul, South Korea; 6 Department of Ophthalmology, Otorhinolaryngology and Dermatology of Korean Medicine, College of Korean Medicine, Kyung Hee University 26723, Seoul, South Korea; 7 Department of Anatomy & Information Sciences, College of Korean Medicine, Kyung Hee University 26723, Seoul, South Korea; Jining Medical University, Jining, China

**Keywords:** Atopic dermatitis, gut microbiome, acupuncture treatment, therapeutic responsiveness

## Abstract

The gut microbiome is increasingly recognized as a central regulator of immune homeostasis, metabolic balance, and therapeutic outcomes. Atopic dermatitis (AD), a chronic inflammatory skin disease, is closely linked to gut microbial dysbiosis. Traditionally regarded as a neurostimulatory therapy, acupuncture (Acu) has demonstrated increasing efficacy in alleviating AD symptoms and improving gastrointestinal function. These observations suggest that the therapeutic effects of Acu in AD may be mediated, in part, by modulation of the gut microbiome. In this study, AD patients were stratified into responder (R) and non-responder (NR) groups based on clinical improvement. Gut microbiome profiling revealed that R patients exhibited greater microbial diversity and compositional stability, indicative of a more balanced gut ecosystem. Specific taxa, including *Alistipes ihumii* and *Odoribacter splanchnicus*, were enriched in R individuals and may serve as microbial predictors of treatment responsiveness. Importantly, fecal microbiota transplantation (FMT) from R donors restored Acu efficacy in a mouse model of AD, whereas FMT from NR donors did not. These findings support the gut-skin axis and highlight the integral role of the gut microbiome in mediating the therapeutic effects of Acu for AD, suggesting potential for microbiome-based personalized treatment.

**IMPORTANCE:**

Increasing evidence supports the gut microbiome’s role in modulating treatment responses in atopic dermatitis (AD), but direct evidence linking acupuncture efficacy with microbiome composition has been lacking. Previous studies did not assess causal relationships via fecal microbiota transplantation (FMT) or functional metagenomics. This study identifies specific gut microbes associated with acupuncture response in AD and confirms their causal role using FMT. It also links functional metabolic pathways to therapeutic efficacy, offering a mechanism-based insight. Our findings support microbiome-informed personalized acupuncture approaches for AD and suggest gut microbiota as a therapeutic modulator in neuroimmune regulation.

**CLINICAL TRIALS:**

This study was registered in the Korean Clinical Trial Registry (CRIS, registration number: KCT0005422).

## INTRODUCTION

The human gut microbiome is essential for maintaining overall health, and its composition significantly differs between healthy individuals and those with diseases (1). This complex gut microbial community contributes not only to digestive processes but also to immune regulation, metabolic activities, and neurological functions. Increasing evidence indicates that the gut microbiome also acts as a critical modulator of treatment outcomes across a wide spectrum of health conditions. For instance, variations in gut microbiome composition and activity have been shown to influence therapeutic responses in cancer (2, 3), hematologic malignancies (4), depressive disorder (5), gallstone disease (6), metabolic syndrome (7), and irritable bowel syndrome (8). In general, patients with higher treatment efficacy tend to have more diverse gut microbiota and greater abundance of microbes capable of producing metabolites that mitigate disease severity.

Neural stimulation has emerged as a promising therapeutic approach to alleviate symptoms and foster recovery by modulating neuroimmune responses, alleviating pain, and regulating neurotransmitter and neurohormone production (9–15). Among various neural stimulation methods, acupuncture (Acu) is an effective therapeutic approach that modulates neural activity by activating peripheral nerves in the skin and muscles, thereby initiating signal transmission to the central nervous system (14, 15). These nerve signals are processed in brain regions, particularly those involved in pain and itch control (16), leading to the release of neurotransmitters that alleviate pain and regulate physiological functions (17–19). The gut microbiome also contributes to the responsiveness to neurostimulation-based treatments. It influences the gut-brain axis signaling and is involved in the production of neurotransmitters, which may enhance or modulate the effects of neurostimulation therapies (20, 21).

Atopic dermatitis (AD) has shown notable therapeutic responsiveness to Acu treatment (22–24). AD is a chronic inflammatory skin disease characterized by severe itching, xeroderma of the skin with dryness and roughness, eczema, and psychological distress in severe cases, such as depression and insomnia (25).

In addition, with the increasing number of AD patients reporting gastrointestinal symptoms, an association between AD and leaky gut syndrome was demonstrated (26, 27). Differences in microbial richness and diversity between healthy individuals and AD patients further underscore the relevance of the gut microbiome in AD pathophysiology. Notably, Acu treatment has shown enhanced efficacy in AD patients with gastrointestinal symptoms (28), and their gut microbiome profiles differ from those without gastrointestinal symptoms (29). Such evidence collectively suggests a potential link between neurostimulation and gut microbiota, positioning Acu treatment in AD as a particularly informative model for investigating gut microbiota-neurostimulation interactions.

The objective of this study is to elucidate the role of the gut microbiota in modulating the therapeutic efficacy of Acu treatment in AD patients. We addressed this by comparing gut microbial diversity, compositional resilience, and key taxonomic features, as well as their associations with predicted metabolite profiles, in patient groups exhibiting high versus low therapeutic responsiveness. To functionally validate the impact of microbial differences between the two groups, a fecal microbiota transplantation (FMT) approach was employed in an AD mouse model. These investigations could provide mechanistic insights into how the gut microbiome synergistically interacts with neuromodulation therapies, with implications for more targeted, microbiome-informed treatment strategies.

## MATERIALS AND METHODS

### Study design and specimen collection

Patient recruitment and all trial processes were carried out at Kyung Hee University Korean Medicine Hospital. One certified Korean Medicine Doctor (KMD) screened patients based on the following inclusion/exclusion criteria and confirmed their voluntary consent for participation in the clinical study if they were eligible. The inclusion criteria are as follows. AD was diagnosed in accordance with the Hanifin and Rajka criteria, and participants had scores ranging from 10 to 40 points on the objective scoring of atopic dermatitis (SCORAD) scale. The exclusion criteria are as follows. Individuals who had taken drugs, particularly antibiotics, or functional foods for health, particularly probiotics and prebiotics, within 1 month before study participation, as these could potentially affect the study results, were excluded. Individuals who had a history of alcohol consumption within 1 week before study participation were excluded. Individuals who had been diagnosed with other diseases, such as neuropsychiatric disorders, brain surgery history, stroke, epilepsy, dementia, or alcohol or drug use disorder, which may affect the results, were excluded. Individuals who had been diagnosed with somatosensory-related diseases, such as Raynaud’s disease or chronic pain disease, were excluded. Individuals who smoked were excluded.

### Acupuncture treatment

Patients who were screened subsequently received Acu treatment. The study was conducted according to the STRICTA 2010 guidelines. Acu was performed by two KMDs, each with at least 2 years of clinical experience and over 10 h of training to ensure standardized procedures. The Acu treatment sessions were held twice a week for 4 weeks, targeting specific acupoints with disposable sterile needles (0.25 × 40 mm; Dongbang Acupuncture Inc., Bundang, Seongnam, Korea): PC6, LI11, and ST36 on both sides, and GB41, ST43, and LI3 on only one side. Needle insertion depth ranged from 5 mm to 30 mm, and needles remained in place for 15 min.

The randomized controlled trial (RCT) protocol of this clinical study can be found in more detail in the following paper (30). This RCT evaluated AD patients in two aspects: skin symptoms and blood inflammation indicators. AD symptoms were assessed using the SCORAD, eczema area, and severity index (EASI). The primary endpoint, changes in SCORAD, was analyzed using a mixed-effects model for repeated measures to account for within-subject correlation and to handle missing data appropriately. Blood inflammation level indicators were investigated by analyzing blood samples for thymus and activation-regulated chemokine (TARC) and autotaxin levels. These evaluations were conducted at week 0 (baseline), week 2 (midterm), and week 4 (endpoint). The study outcomes were assessed by an investigator blinded to the treatment assignments.

### Responder (R) vs non-responder (NR) group selection criteria

**Fig 1:**
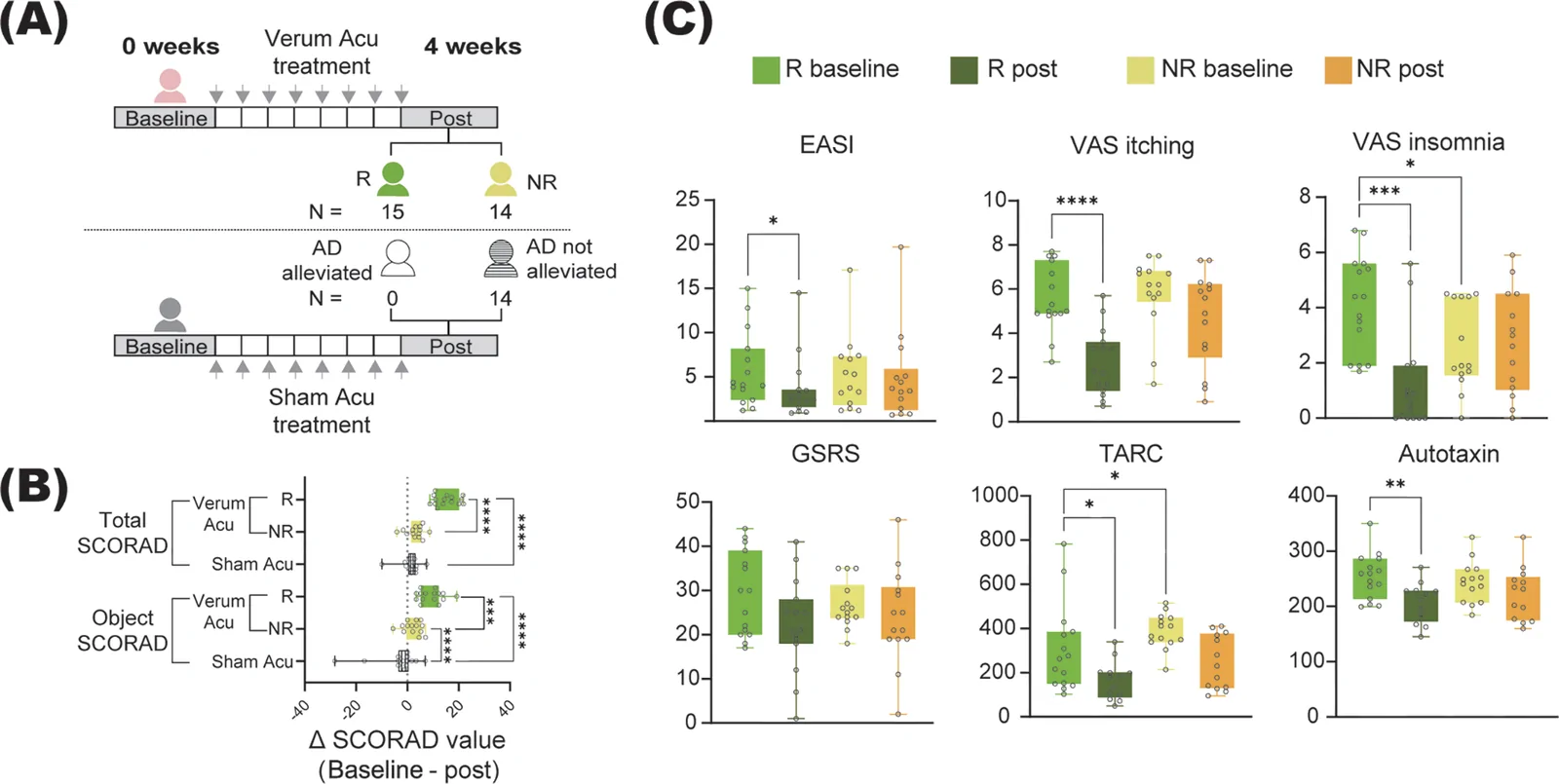
Clinical indices in AD patients enrolled in the study. (**A**) Overview of studied patients. (**B**) Changes in SCORAD (∆SCORAD) before and after Acu treatment in the verum Acu R, verum Acu NR, and sham Acu groups. (**C**) Clinical inflammatory indicator values in verum Acu patients. “Baseline” and “post” indicate values measured before and after verum Acu treatment, respectively. “Baseline” matched samples before Acu treatment (pre-samples). When comparing parameters from two categories, the Mann-Whitney *U*-test was used. *P-value <0.05, **P-value <0.01, ***P-value <0.001, ****P-value <0.0001; Acu, acupuncture; R, responder group; NR, non-responder group; EASI, eczema area and severity index; VAS, visual analog scale; GSRS, gastrointestinal symptom rating scale; TARC and autotaxin, pg/mL.

SCORAD is a widely used clinical tool for assessing the severity of AD. It consists of total SCORAD, which takes into account patient-reported symptoms such as itching and insomnia, and objective SCORAD, which focuses on observable factors like lesion extent and intensity. AD patients were classified into the R and NR groups based on a total SCORAD change value of 8.7 and an objective SCORAD change value of 8.2 before and after Acu treatment. The threshold of 8.7 points corresponds to the minimal clinically important difference (MCID) reported in a previous study (31). Therefore, after completing the Acu treatment sessions and outcome assessments, participants were divided into the R group with higher treatment efficacy and the NR group with no treatment effect based on their delta (∆) SCORAD values according to the MCID. For the control group, participants were assigned to the NR group after confirming that there was no clear change in their SCORAD scores. While objective SCORAD reflects physician-assessed clinical signs, total SCORAD also includes subjective symptoms such as pruritus and sleep disturbance. In this context, only the R group showed significant improvement in both measures, indicating a more comprehensive clinical response. Our data demonstrate a clear separation between the two groups regarding treatment responsiveness. Specifically, the mean ΔSCORAD for the R group was approximately 15.1 (decreasing from 37.2 to 22.1), which significantly exceeds the 8.7 MCID threshold. In contrast, the NR group exhibited a mean change of only 3.1 (from 29.4 to 26.3). Furthermore, the individual distribution illustrated in Fig. 1B confirms that very few participants were positioned near the threshold boundary, thereby ensuring a robust and reliable classification of treatment response.

### Gut microbiome data analysis

#### 16S rRNA gene sequencing and analysis

Participants self-collected fecal samples from the central part of the stool using sterile 4N6FLOQSwabs Genetics or FLOQSwabs (Copan Diagnostics, Brescia, Italy). DNA was isolated from fecal swab samples using the DNeasy PowerSoil Pro Kit (Qiagen, Hilden, Germany) according to the manufacturer’s instructions. The V3-V4 hypervariable regions of the 16S rRNA gene were targeted using a universal primer set (5′-CCTACGGGNGGCWGCAG and 5′-GACTACHVGGGTATCTAATCC) with sequencing barcodes. Sequencing was performed on an Illumina MiSeq platform with a 2 × 250 bp paired-end protocol, yielding paired-end reads. We mainly followed the QIIME2 (32) pipeline for bacteria profiling based on 16S rRNA. DADA2 (33) was selected as a tool for sequence quality control, and the SILVA (34) database was chosen as the taxonomic reference database. The sequence data obtained from the V3-V4 region of the 16S rRNA gene in 29 fecal microbiota samples revealed the presence of 261 genera, 573 species, and 1,821 ASVs. The results were imported into the R statistical environment for further analysis using the Bioconductor package phyloseq. Additionally, generated taxonomy tables, phylogenetic trees, and metadata were imported into the R environment for incisive microbial analyses, for further analysis or visualization. The number of observed ASVs, Shannon and Faith PD, and inverse Simpson diversity indices were selected as alpha diversity indicators. Beta diversity was determined using unweighted UniFrac phylogenetic distance matrices. The Bray-Curtis dissimilarity index assessed gut microbiota variability to understand compositional changes pre- and post-treatment. The dysbiosis score was analyzed using the unweighted UniFrac phylogenetic distance between the R and NR groups before and after Acu treatment using the euclideanDistCentroids function in the dysbiosisR R package.

#### Metagenome shotgun sequencing and analysis

Eleven fecal samples from AD patients, subjected to 16S rRNA amplicon sequencing, were selected for whole metagenome sequencing (WMS) to validate and complement the 16S-based findings, with sample selection designed to maintain a demographic and clinical profile representative of the full cohort (Table S1). Paired-end sequencing for WMS was performed on an Illumina MiSeq platform. Using already known public data information as a reference, we profiled the data by aligning them with our data. Taxonomic features were obtained by using MetaPhlAn v.4.0 (35), and functional features were obtained by using HUMAnN v.3.0 (36).

Additionally, metagenome-assembled genomes (MAGs) were analyzed based on our data to obtain genome information of high quality. The metagenome assembly was conducted using MEGAHIT, which uses the process of selecting the k-mer size. Metagenome binning was conducted using METABAT2 v.2.15 to make MAGs. To check MAGs’ quality and align taxonomy information, MAGs with 20% completeness and contamination of <10% were selected with CheckM v.1.2.2. Average nucleotide identity (ANI) was calculated for selected MAGs. For each sample, dRep was run to obtain the highest-quality genome from the genome with 99% ANI. Genes were predicted on contigs (from the co-assembly and from the individual assemblies) using Prodigal v.2.6.3. Through Prodigal, the format of FAA was obtained. Functional annotation of proteins was conducted using EggNOG v.2.1.12. Annotations against EggNOG were performed using eggNOG-mapper.

#### Quantification analysis

Receiver operating characteristic (ROC) and area under the curve (AUC) analyses were performed using bacterial relative abundance to identify microbial markers distinguishing the R and NR groups. Microbial features with an AUC ≥0.7 were selected as candidate markers for group classification, using the R package ROCR for performance evaluation. The R package randomForest was used to perform random forest regression to model the gut microbiota R group based on the relative abundance of bacterial genera obtained from amplicon sequencing of 29 pre-Acu treatment samples. To evaluate model performance and ensure internal validity, out-of-bag (OOB) estimation was applied, which is an inherent cross-validation procedure in the random forest algorithm whereby each tree is validated on samples not used during its construction. The OOB error rate was calculated as a measure of generalization performance. Additionally, for ROC analyses of individual microbial features and clinical variables, no additional resampling was applied given the exploratory nature of the analysis; AUC values are reported as descriptive performance metrics. The linear discriminant analysis effect size (LEfSe) (37) was used as a method for assessing differential taxonomic and functional abundances in AD patients before Acu treatment. DESeq2 analysis was carried out on FMT-experimented fecal samples utilizing read counts from WMS.

Spearman correlation analysis was performed with the relative abundance of 16S rRNA sequencing in AD patients before Acu treatment to cluster significantly correlated genera in 16S rRNA amplicon sequencing. Then, before Acu treatment, patients with AD were subjected to Spearman correlation analysis only with genera’s relative abundance to derive the cluster with the highest number of edges. All correlation analysis were performed using R Statistical Software (v.4.3.1)

### Human microbiota fecal transplantation

#### Human microbiota transplantation into antibiotics-treated conventional mice

Six-week-old male C57BL/6NTac mice were obtained from DBL (Korea) and maintained under standard housing conditions of a 12 h light:dark cycle with free access to food and water. All animals were used in experiments after a 7 day acclimatization period. All cages, water bottles with stoppers, drinking water, food, and bedding were autoclaved before being used in the experiment.

To deplete gut microbiota, the mice were pretreated with 200 µL of a broad-spectrum antibiotics cocktail containing 1 mg/mL ampicillin, 1 mg/mL gentamicin, 1 mg/mL neomycin, 1 mg/mL metronidazole, and 0.5 mg/mL vancomycin via oral gavage once a day for 5 days, followed by a 2 day washout. Fresh feces were collected from them pre-treatment, mixed thoroughly in sterile saline (0.1 g/mL), and thereafter centrifuged at 1,000 rpm for 5 min to eliminate particulate matter. The resultant suspension was centrifuged at 8,000 rpm for 5 min at 4°C to obtain total bacteria. The precipitates were mixed with a final concentration of 20% sterile glycerol and stored at −80°C. On the day of FMT, the suspensions were individually thawed, briefly mixed, and diluted to an OD at 600 nm of 0.5 in sterile saline, corresponding to approximately 10^8^ CFU/mL. A total of 200 µL of the bacterial suspension from either R or NR donors was gavaged into recipient mice, with each donor sample delivered to 8 mice, once a day for 5 consecutive days.

#### MC903-induced AD development and Acu treatment

The R or NR recipient mice were then subsequently given topical application of MC903 (1.65 μg in 20 μL ethanol; Sigma-Aldrich) once a day to the cheek for 7 consecutive days, followed by a 4 day post-treatment period. Acu was administered once daily at the bilateral Gok-Ji (LI11) acupoints for 11 days (the 7 day MC903 treatment and the 4 day post-treatment period). The LI11 acupoint is located in the dip on the lateral end of the cubital crease when the elbow is fully flexed (25). LI11 was chosen because it is a common acupoint used to treat eczema (38), and we have previously demonstrated the efficacy of Acu at this acupoint in treating AD-related inflammation and itch (25). Mice were gently grasped by hand and treated with small Acu needles (0.18 × 8 mm; Haenglim-Seoweon Acuneedle Co., Korea) inserted to a depth of 2 mm–3 mm. Each LI11 point was manually stimulated by twisting for 30 seconds at a rate of twice per second, after which the needles were immediately withdrawn..Mice that did not receive Acu were also slightly grabbed by hand for 60 s without inserting a needle.

#### Skin lesion severity

The severity of AD-like skin lesions on day 11 after the initial application of MC903 was graded on a 0–3 scale (0 = none; 1 = mild; 2 = moderate; 3 = severe) for redness (erythema), dryness (xerosis), and scabbing (excoriation), and the scores were summed. Lesion scoring was performed in a blinded manner.

#### Histological evaluation

Lesioned skin tissues were fixed in buffered 10% formalin, embedded in paraffin, cut into 5 μm-thick sections, deparaffinized, and stained with hematoxylin-eosin, toluidine blue, and Congo red. Evaluation of sections was conducted under a light microscope with 100× magnification for quantitative analysis. Epidermal hyperplasia was determined by measuring the epidermal thickness of the lesional skin in four to five areas per tissue section using ImageJ software. Mast cells were quantified by counting violet-stained cells in toluidine blue-stained lesional skin sections, and eosinophils by counting red granules in Congo red-stained sections. All histological evaluations were conducted in a blinded fashion.

#### Immunofluorescence staining

Immunofluorescence staining was performed on 5 μm-thick sections obtained from formalin-fixed, paraffin-embedded tissue. After deparaffinization, sections were antigen retrieved by boiling the sections in citrate buffer (pH 6.0), permeabilized with Triton X-100 (0.1%), blocked in 3% bovine serum albumin in PBST, and then incubated with a primary antibody against thymic stromal lymphopoietin (TSLP) (1:200; GeneTex) for 72 h at 4°C, followed by Alexa Fluor 594-conjugated secondary antibody (1:300, Invitrogen Molecular Probes) for 1 h. Finally, the sections were mounted using Vectashield Antifade Mounting Medium with DAPI (Vector Labs). Photos were taken with a Zeiss Axio Imager M2 fluorescence microscope (Carl Zeiss), and fluorescence intensities were quantified using Zeiss ZEN 3.8 software (Carl Zeiss) in a blinded fashion. Slices with normal IgG as a primary antibody were used as a negative control.

### Statistical analysis

In all statistical analyses comparing two groups, a *t*-test was performed when the data followed a normal distribution, and a Mann-Whitney *U*-test was used when the data did not follow a normal distribution. Statistical multi-group comparisons were performed by one-way ANOVA with Tukey’s multiple comparison test. The *t*-test, Mann-Whitney *U*-test, and one-way ANOVA with Tukey’s multiple comparison test were performed using GraphPad Prism 10.2. Furthermore, the permutational multivariate analysis of variance was performed with 999 permutations using the adonis function in the vegan R package. A *P*-value <0.05 was considered statistically significant.

### Role of funders

The sources of funding did not influence the design of the study, the collection of data, the analysis of data, the interpretation of results, or the writing of the manuscript.

## RESULTS

### Acupuncture-induced neuromodulation alleviates AD symptoms with responders showing notable improvement

A total of 43 participants who met the inclusion criteria were enrolled in the study (Fig. 1A and Fig. S1). These participants received eight Acu treatment sessions over a 4-week period, and all completed the treatment without any dropouts. Baseline data collection, including health assessments and the collection of blood and stool samples, was conducted prior to the treatment phase. Participants were randomly assigned to either the verum Acu group, in which needles penetrated the skin, or the sham Acu group, in which non-penetrating pressure was applied to the same acupoints (39).

Verum Acu treatment, received by 29 participants, resulted in a significant reduction in both total and objective SCORAD scores compared to the sham Acu group of 14 participants (each *P* < 0.005, ***), supporting the neuroimmune modulatory effects of Acu in AD (Fig. 1B). In contrast, the sham Acu group showed no notable improvement, further suggesting that Acu-induced neuromodulation plays a key role in alleviating AD symptoms. There was no significant difference in baseline characteristics between the verum and sham Acu groups (Table S2). While clinical improvement was observed in the verum Acu group, individual responses varied, with participants classified as R and NR. Based on the MCID, participants with a ∆SCORAD ≥8.7 were classified as the R group, indicating an effective response to verum Acu treatment, whereas participants with a ∆SCORAD <8.7 were classified as the NR group. Based on this criterion, the 29 participants who received verum Acu were divided into R (*n* = 15) and NR (*n* = 14) groups. Compared to the R group, the NR group within the verum Acu treatment and the sham Acu group both did not show any significant improvement in total SCORAD, indicating symptom relief was specific to those who responded to verum Acu. Accordingly, given that this study aims to investigate the specific therapeutic effects of Acu and their relationship with the gut microbiome, subsequent analyses were restricted to participants in the verum Acu group.

Among the objective clinical measures used to evaluate AD symptoms, EASI scores significantly decreased in the R group following Acu treatment, indicating objective clinical improvement. Subjective symptom assessment using the visual analog scale (VAS) also showed significant improvement in the R group. Notably, baseline insomnia scores were already higher in the R group compared to the NR group. The gastrointestinal symptom rating scale (GSRS) did not differ significantly between the R and NR groups, but within the R group, a trend toward symptom improvement was observed following Acu treatment. Regarding inflammatory biomarkers, both TARC and autotaxin levels significantly decreased in the R group after Acu treatment. In addition, baseline TARC levels were significantly higher in the R group compared to the NR group (Table 1 and Fig. 1C). Taken together, these results imply that Acu may primarily act by reducing the inflammatory burden underlying AD, while subjective symptom relief, such as pruritus and insomnia, may depend on additional individual-specific factors.

**TABLE 1:** Clinical characteristics and indices of the R and NR groups who received verum Acu treatment^*a*^

		R	NR	*P*-value
		(*N* = 15)	(*N* = 14)	
Age		24.3 ± 3.5	25.6 ± 8.0	0.559
Sex				0.329
	Male	7 (46.7%)	10 (71.4%)	
Height		168.1 ± 8.4	168.8 ± 9.6	0.826
Weight		65.6 ± 13.4	68.6 ± 12.9	0.555
BMI		23.0 ± 3.2	23.9 ± 2.8	0.448
BDI		25.5 ± 3.4	26.2 ± 4.7	0.654
GSRS				
	Baseline	29.3 ± 9.6	26.6 ± 5.3	0.355
	Post	22.1 ± 10.5	24.0 ± 10.8	0.641
VAS itching				
	Baseline	5.6 ± 1.5	5.8 ± 1.7	0.742
	Post	2.7 ± 1.5	4.6 ± 2.1	0.008
VAS insomnia				
	Baseline	4.1 ± 1.8	2.6 ± 1.6	0.019
	Post	1.2 ± 1.8	2.7 ± 1.9	0.032
SCORAD total				
	Baseline	37.2 ± 8.4	29.4 ± 7.9	0.017
	Post	22.1 ± 7.7	26.3 ± 8.1	0.165
SCORAD object				
	Baseline	27.5 ± 8.5	21.0 ± 6.8	0.033
	Post	18.2 ± 7.3	18.9 ± 7.0	0.798
EASI				
	Baseline	5.7 ± 4.2	5.3 ± 4.2	0.773
	Post	3.7 ± 3.5	4.9 ± 5.0	0.447
TARC (pg/mL)				
	Baseline	304.5 ± 197.7	387.4 ± 79.8	0.151
	Post	164.1 ± 85.1	229.7 ± 134.8	0.147
Autotaxin (pg/mL)				
	Baseline	254.2 ± 41.4	244.0 ± 37.8	0.497
	Post	205.4 ± 36.2	206.6 ± 75.3	0.957
DLQI				
	Baseline	8.4 ± 5.0	9.6 ± 4.1	0.472
	Post	3.8 ± 2.8	6.6 ± 3.3	0.021
Credibility test				
	Baseline	18.0 ± 3.8	17.5 ± 2.9	0.694
	Post	19.7 ± 2.4	15.9 ± 4.5	0.011

^*a*^ Abbreciations: BMI, body mass index; BDI, beck depression inventory; GSRS, gastrointestinal symptom rating scale; VAS, visual analog scale score; SCORAD, scoring atopic dermatitis; EASI, eczema area and severity index; TARC, thymus and activation-regulated chemokine; DLQI, the dermatology life quality index; R, responder; NR, non-responder.

### Compared to non-responders, responders maintained a more stable gut microbiota composition throughout the Acu treatment period

**Fig 2:**
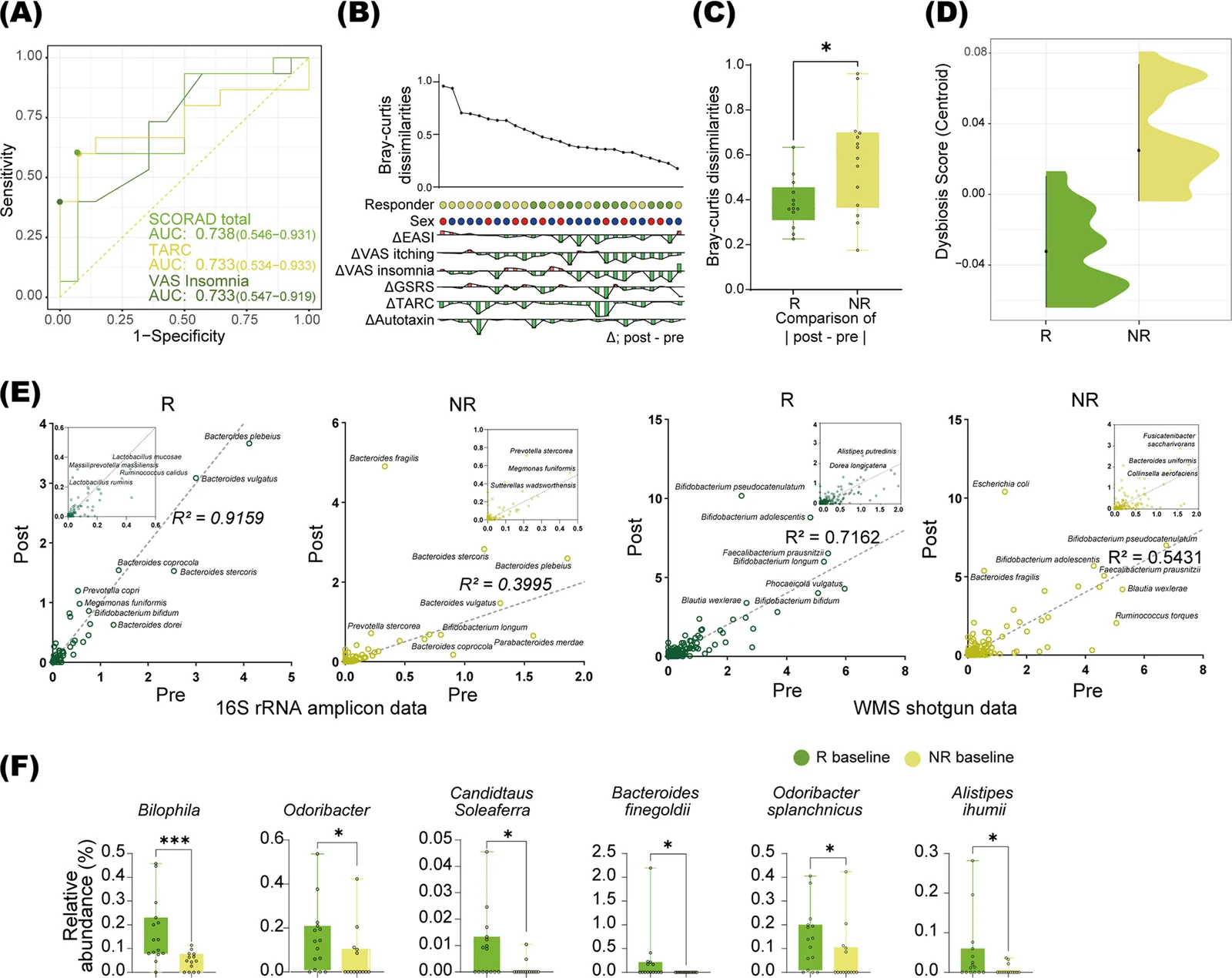
Microbial composition and clinical indices distinguish acupuncture responders from non-responders in AD patients. (**A**) ROC curves of clinical markers measured in baseline-treated samples to identify key indicators associated with gut microbiota composition differences between R and NR; only those with AUC >0.7 are shown. (**B**) Samples are ordered by decreasing Bray-Curtis dissimilarity values representing the magnitude of gut microbiota compositional change between baseline- and post-treatment. Circles indicate treatment response (green: R, yellow: NR) and gender (blue: male, red: female). Bar plots show delta (Δ; post-baseline) values for clinical and inflammatory indicators, including EASI, VAS itching, VAS insomnia, GSRS, TARC, and autotaxin. (**C**) Comparison of gut microbiota compositional changes between baseline- and post-treatment samples in R and NR groups, as measured by Bray-Curtis dissimilarity. (**D**) Dysbiosis scores based on unweighted UniFrac distances between R and NR groups. (**E**) Correlation of species-level relative abundance in baseline R or NR (*x*-axis) against species-level relative abundance in post R or NR (*y*-axis), based on both 16S rRNA amplicon and WMS data. (**F**) Relative abundance of discriminatory taxa *Bilophila*, *Bacteroides finegoldii*, *Odoribacter splanchnicus*, *Alistipes ihumii*, *Bacteroides,* and *Candidatus Soleaferra* using LefSE analysis. Statistical significance assessed by the Mann-Whitney *U*-test. *P-value <0.05; ***P-value <0.001. ‘Baseline’ matched samples before Acu treatment (pre-samples).

Building upon our clinical findings that the R group experienced greater symptom relief following Acu treatment, we next investigated whether distinct gut microbial community structures were associated with this therapeutic responsiveness. We hypothesized that individuals in the R group would exhibit greater gut microbiota stability, indicative of more robust microbial homeostasis, compared to those in the NR group. To test this, 16S rRNA gene sequencing was performed on fecal samples from verum Acu-treated AD patients. Of these, 11 participants were additionally selected for higher-resolution WMS. Before evaluating microbial shifts, we first sought to identify which clinical variables were most strongly associated with gut microbiota composition. For this purpose, the AUC was calculated using microbial abundance and clinical metadata. Among these, total SCORAD emerged as the most distinguishing factor between the R and NR groups. Additionally, TARC and VAS insomnia were also identified as key differentiating variables based on microbiome profiles (Fig. 2A). These results align with the clinical findings, as both TARC and VAS insomnia exhibited significant symptom changes in the R group following Acu treatment. To assess the internal validity of the random forest model, OOB estimation was employed. The OOB error rate was 17.9% (OOB accuracy = 82.1%), indicating that the model distinguished responders from non-responders with reasonable generalizability, even without an independent test set. This level of classification performance is considered acceptable given the high-dimensional and sparse nature of microbiome data. To quantify individual-level microbiota shifts induced by Acu, Bray-Curtis dissimilarity was used to measure the pre- to post-treatment change for each participant, ranked from highest to lowest (Fig. 2B). A negative ∆EASI value, reflecting symptom relief, tended to align with lower Bray-Curtis dissimilarity, suggesting minimal microbiota perturbation in clinical responders.

A scatter plot of α-diversity revealed that the R group tended to have greater microbial richness and evenness (Fig. S2). Microbial shifts were more pronounced in the NR group, as indicated by a significant increase in Bray-Curtis dissimilarity compared to the R group. In contrast, the R group exhibited minimal intra-individual compositional changes between pre- and post-Acu treatment samples, reflecting greater microbial stability (Fig. 2C). Here, microbial stability is defined as a lower intra-individual compositional shift over the treatment period, as quantified by within-subject Bray-Curtis dissimilarity based on relative abundance profiles derived from 16S rRNA amplicon sequencing (40). To more precisely assess the gut microbiome imbalance, a dysbiosis score was calculated, confirming the greater disruption of microbial composition in the NR group (Fig. 2D). To further explore microbiota stability in response to Acu treatment, we compared relative abundance before and after Acu treatment. In the R group, relative microbial composition remained highly consistent between the pre- and post-treatment time points, resulting in *R*² values greater than 0.9. These values, which were significantly higher than those observed in the NR group, reflect the greater stability of the gut microbiome in the R group. (Fig. 2E). Taken together, higher responsiveness to Acu treatment in AD was associated with a more stable gut microbiota composition, as reflected by lower dysbiosis scores and minimal relative compositional differences between pre- and post-treatment samples.

To identify microbial taxa contributing to this stability in response to Acu treatment, we analyzed only pre-treatment fecal samples. This approach was based on the rationale that baseline microbial composition is a key determinant of post-treatment community dynamics and may influence the resilience of the gut microbiome. By focusing on pre-Acu samples, we aimed to uncover microbial features that distinguish R from NR patients and potentially support microbial homeostasis during and after Acu treatment. The statistical analysis using LefSE was performed to identify microbial taxa that differed between the R and NR groups before Acu treatment. As shown in Fig. 2F, these six microbial taxa exhibited significantly higher relative abundance in the R group compared to the NR group prior to Acu treatment. To investigate potential relationships among the microbial taxa, a network was constructed showing only significant correlations (*P*-value <0.05) based on Spearman correlation (Fig. S3). Core clusters were located at the center of the network and exhibited the highest number of edges. Although *Odoribacter* is absent from the network due to the non-significant *P*-value, *Odoribacter* and *Alistipes* are in the same core cluster. Given its enrichment in R-associated biomarkers, the core cluster may function as a structural hub that contributes to the stability of the gut microbiota in the R group.

### AD mice receiving microbiota from responders exhibit enhanced therapeutic response to acupuncture treatment

**Fig 3:**
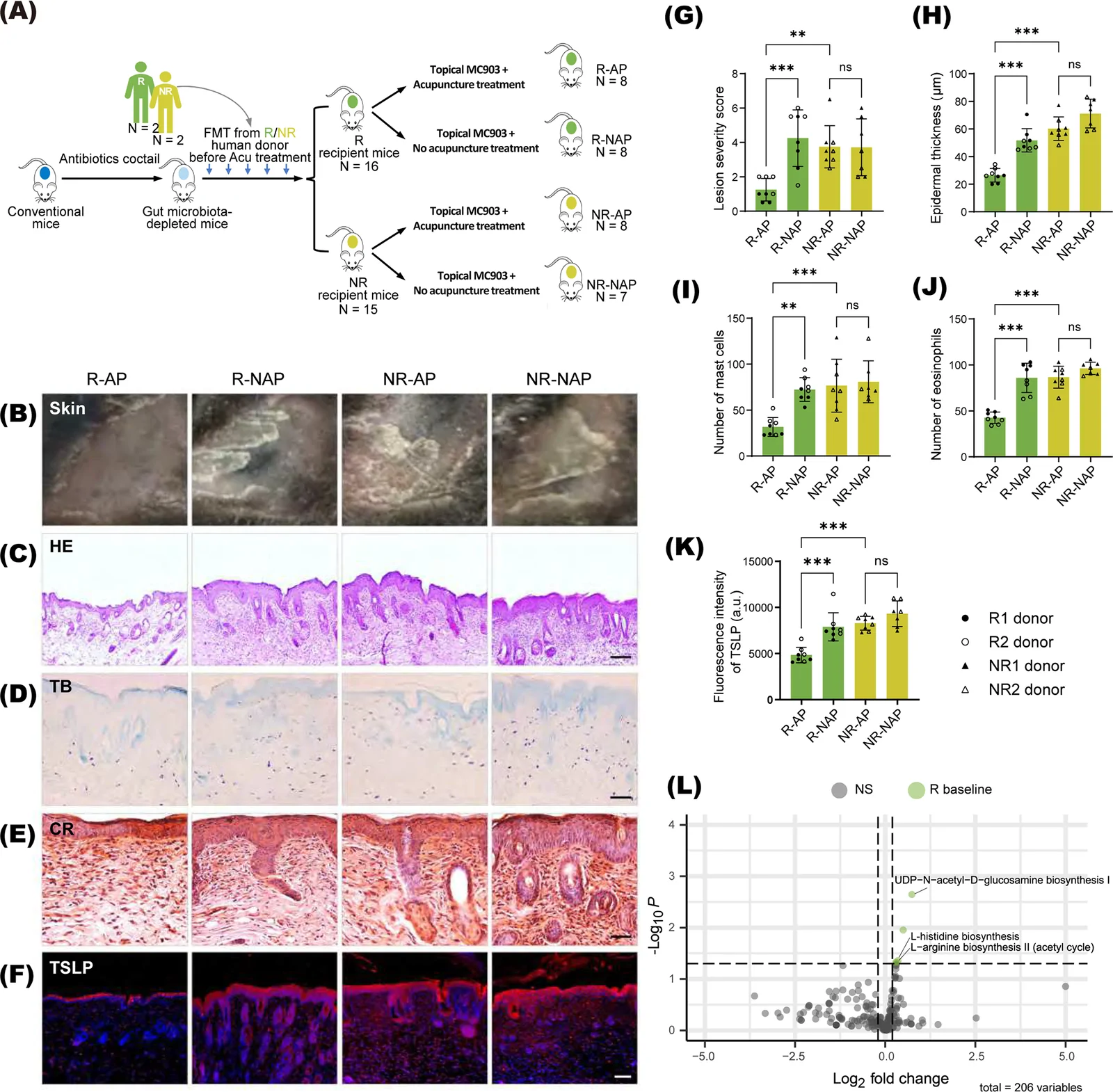
Gut microbiota derived from responders mediates therapeutic efficacy of acupuncture in the atopic dermatitis mouse model. (**A**) Schematic of the experimental design for FMT from R or NR patients into antibiotic-treated conventional mice, followed by MC903-induced AD and Acu treatment. (**B**) Representative pictures of skin manifestations of the indicated mice. (**C**) Representative hematoxylin and eosin (HE) staining of lesional skin from indicated mice. Scale bars, 100 μm. (**D**) Representative toluidine blue (TB) staining of lesional skin from indicated mice for mast cell evaluation. Scale bars, 100 μm. (**E**) Representative Congo red (CR) staining of lesional skin from indicated mice for eosinophil evaluation. Scale bars, 50 μm. (**F**) Representative confocal immunofluorescence images of lesional skin from the indicated mice, stained with TSLP (red) and counterstained with DAPI (blue). Scale bars, 100 μm. (**G**) Skin lesion severity scores shown in panel **B**. (**H**) Measurement of the epidermal thickness shown in panel **C**. (**I**) Quantitation of mast cells shown in panel **D**. (**J**) Quantitation of eosinophils shown in panel **E**. (**K**) Quantitation of fluorescence intensities of TSLP shown in panel **F**. (**L**) Volcano plot showing differential abundance analysis of microbial metabolic pathways in fecal samples from the FMT experiment comparing the R and NR groups. Green dots represent pathways significantly enriched in the R group (*P* < 0.05 and |log₂FC| > threshold). Data represent mean ± SD. Statistical significance was assessed by one-way ANOVA with Tukey’s multiple comparison test. NS, not significant (*P* ≥ 0.05); *P < 0.05, **P < 0.01, ***P < 0.001.

We next conducted FMT to determine whether gut microbiota play a causal role in determining Acu treatment responsiveness. Fecal samples collected from R or NR patients prior to Acu treatment were transferred into antibiotic-treated mice via oral gavage. Following FMT, mice were subjected to MC903-induced AD development and Acu treatment (Fig. 3A). R donor samples exhibited markedly higher levels of R-enriched taxa (Fig. S4). To first validate the therapeutic efficacy of Acu in MC903-induced AD mice, we assessed clinical symptoms and inflammatory parameters, including skin severity, epidermal hyperplasia, infiltration of mast cells and eosinophils, and TSLP expression in lesional skin. Acu treatment of responder recipient mice (R-AP mice) effectively decreased the noticeable skin lesions induced by MC903 compared to their non-acupuncture-treated counterparts (R-NAP mice), whereas no significant effect was observed in non-responder recipient mice treated with acupuncture (NR-AP mice) compared to their non-acupuncture-treated counterparts (NR-NAP mice; Fig. 3B and G). Consistent with this finding, Acu treatment significantly improved epidermal hyperplasia, as indicated by reduced epidermal thickness, in R recipient mice, but not in NR recipient mice (Fig. 3C and H). Acu treatment was also observed to reduce the infiltration of mast cells and eosinophils into AD-like lesional skin in R-recipient mice, but not in NR recipients (Fig. 3D and E). The numbers of mast cells and eosinophils in the dermis of Rr-AP mice were significantly reduced compared to those in Rr-NAP mice, while no difference was observed between NR-AP and NR-NAP mice (Fig. 3I and J). Moreover, immunofluorescence staining analysis revealed that expression levels of TSLP, a trigger factor for the pathogenesis of AD, were significantly suppressed in the epidermis of lesional skin of R-AP mice compared to R-NAP mice, whereas no significant differences were observed between NR-AP and NR-NAP mice (Fig. 3F and K). Altogether, these results demonstrate that microbiota from R individuals are sufficient to transfer therapeutic responsiveness to Acu in a murine AD model, whereas NR microbiota are not.

To determine which microbial metabolites are associated with Acu responsiveness, we compared donors from the R and NR groups and identified five microbial metabolic pathways that were significantly more abundant in the R donors (Fig. 3L). These included UDP-*N*-acetyl-D-glucosamine biosynthesis I, L-histidine biosynthesis, and L-arginine biosynthesis II. Each of these pathways has been implicated in immune modulation. We then quantified the abundance of genes associated with the certain enriched metabolic pathways in each MAG (Fig. S5A and B). These results suggest that the gut microbiota of Acu responders are not only compositionally distinct but also functionally enriched in metabolite pathways with known immunomodulatory roles.

## DISCUSSION

In this study, we classified treatment responsiveness based on the MCID of the SCORAD index (8.7 points) (31), which is a validated and comprehensive tool for assessing the severity of AD. Although SCORAD was the primary criterion for grouping, our analysis confirms that this classification is not one-dimensional but reflects a broader physiological and immunological recovery. Specifically, the R group, defined by the SCORAD threshold, also exhibited statistically significant improvements in other independent objective measures. For instance, EASI scores significantly decreased in the R group following acupuncture treatment, indicating objective clinical recovery that aligns with the SCORAD-based classification. Furthermore, key inflammatory biomarkers such as TARC and Autotaxin levels showed a significant reduction uniquely in the R group. Given that baseline TARC levels were higher in the R group, these findings suggest that acupuncture may be particularly effective in patients with a specific inflammatory profile. Building on these findings, we further investigated the influence of the gut microbiota on treatment responsiveness to acupuncture and its potential role in modulating therapeutic outcomes in AD patients. The therapeutic outcomes in the present study were achieved through a standardized acupuncture protocol specifically designed to modulate the neuro-immune-skin axis. The selection of the three fixed acupoints—LI11, ST36, and PC6—was based on established clinical evidence and expert consensus. Specifically, LI11 was targeted for its efficacy in reducing lichenification and chronic skin lesions, while ST36 was included for its anti-inflammatory properties, mediated through the inhibition of mast cell degranulation and IL-33 signaling. PC6 served a dual role in symptomatic pruritus relief and autonomic nervous system stabilization. This fixed protocol ensures high reproducibility, and the treatment frequency (twice weekly) was optimized based on our previous pilot trial, which demonstrated that a twice-weekly regimen is as effective as a thrice-weekly one, providing a more feasible and cost-effective parameter for clinical application. As Acu is mainly used to manage pruritus rather than severe inflammation in AD, we focused our study on patients with mild to moderate AD, where inflammatory symptoms are less prominent. Accordingly, most of the enrolled AD patients exhibited non-severe symptoms, with a mean SCORAD score of 32.8. To assess responsiveness to Acu treatment, we classified AD patients into R and NR groups based on SCORAD scores, using the MCID as the criterion. A comparison of the gut microbiome between the R and NR groups revealed that the gut microbiota of AD patients in the R group exhibited greater stability—defined as lower intra-individual compositional shifts over the treatment period (40, 41)—as evidenced by smaller within-subject Bray-Curtis dissimilarity and lower dysbiosis scores between pre- and post-Acu treatment samples. This comparison was critical, as it enabled the identification of microbial features associated with therapeutic responsiveness. Gut microbiome homeostasis (42) is known to support immune function (43, 44), digestion and nutrient absorption (45), and metabolic health (43), which may have contributed to symptom alleviation in the R group. This result suggests that maintaining gut microbiome stability may enhance the effectiveness of Acu treatment and improve outcomes in AD patients. It should be acknowledged, however, that the stability assessed in this study reflects relative compositional change based on 16S rRNA sequencing data and does not capture absolute abundance dynamics of individual taxa. Future studies employing quantitative microbiome profiling approaches would be warranted to further characterize the nature of this stability (40).

The robustness of this classification was further evaluated through a sensitivity analysis using ROC curves. Our data demonstrated that the gut microbiota composition was significantly associated not only with total SCORAD but also with TARC levels and VAS insomnia scores, both achieving an AUC of 0.733. This suggests that the microbial signatures associated with acupuncture responsiveness are consistently linked to both systemic inflammation and subjective symptoms, reducing the potential for bias inherent in a single-metric classification. Collectively, these multifaceted results confirm that the identified microbial-clinical associations are robust and hold up across various grouping criteria and biological levels.

A critical aspect of our study design was the rigorous control for non-specific effects, such as the placebo response or physical handling. While we observed improvements in subjective indices (e.g., VAS for pruritus) across both groups, statistically significant improvements in objective clinical signs (Total SCORAD, Objective SCORAD, and EASI) were uniquely localized to the verum acupuncture group. This discrepancy is consistent with findings that placebo responses frequently manifest in patient-reported subjective outcomes, whereas objective dermatological signs are more sensitive to the specific physiological actions of the intervention. The integrity of this distinction is further supported by the blinding test in our previous study (28) and the credibility assessments in the present study. Initial treatment credibility was identical across groups at baseline (*P*-value = 0.694). These results indicate that the observed objective recovery was not biased by pre-existing expectations or a failure in blinding. Interestingly, the significant post-treatment increases in credibility observed only in the responder group (*P*-value = 0.011) suggest a retrospective reinforcement of trust following actual clinical improvement, which in our study exceeded the MCID of 8.7 points. Collectively, these data reinforce that the therapeutic efficacy of acupuncture in AD is a specific biological phenomenon mediated, at least in part, by the gut-brain-skin axis.

We identified specific gut microbial taxa, such as *Odoribacter splanchnicus* and *Alistipes ihumii*, that were significantly more abundant in the R group before Acu treatment. *O. splanchnicus* is known to produce short-chain fatty acids (46), including acetic, propionic, and succinic acids, which possess anti-inflammatory properties and may contribute to symptom alleviation in AD (47, 48). It has also emerged as a promising next-generation probiotic candidate strain (49), as its outer membrane vesicles (OMVs) have been demonstrated to attenuate intestinal inflammation and enhance gut barrier integrity, a key driver of pro-inflammatory cytokine secretion (50). Consequently, these OMVs exert anti-inflammatory effects by downregulating the pro-inflammatory cytokine IL-8 and upregulating the anti-inflammatory cytokine IL-10 production. Although the role of *A. ihumii* in inflammation is not well understood, its increased prevalence in healthy individuals suggests a potential role in maintaining microbial balance (51). The correlation of *O. splanchnicus* and *A. ihumii* may imply a synergistic role in modulating host-microbe interactions. This suggests that microbial positive interactions may promote ecological resilience within the gut microbiome, enhancing its capacity to accommodate external influences such as acupuncture-induced neuroregulatory effects. Thus, compositional shifts and the community structure of the gut microbiota may contribute to the observed therapeutic outcomes (52).

To directly evaluate whether gut microbiota contribute to Acu responsiveness, FMT was performed. Mice that received fecal transplants from the R donors showed a significant reduction in response to neurostimulation treatment, as evidenced by the marked alleviation of AD-like cutaneous symptoms. In contrast, mice that received fecal transplants from the NR group showed no such effect. These findings provide strong causal evidence that the resident gut microbiota functionally mediate therapeutic responsiveness to Acu treatment, further supported by the suppression of TSLP expression, reduced epidermal thickness, and decreased mast cell infiltration observed exclusively in R-recipient mice. These observations support the functional relevance of R-enriched taxa, and the microbiome is increasingly recognized for its influence on therapeutic responses, including those to cancer therapies (53, 54), extending this paradigm to Acu treatment.

Functional metabolic pathway analysis of R and NR donors used in FMT identified several pathways associated with neurotransmission and inflammation suppression. Among these, L-histidine biosynthesis (55) and L-arginine biosynthesis II (56) were particularly noteworthy due to their relevance in host neuroimmune signaling. Also, the R group harbored significantly more strains encoding L-arginine biosynthesis II, a pathway linked to nitric oxide production and neuronal signaling. Although the mechanisms linking Acu-induced neural stimulation and the gut microbiome remain to be fully elucidated, this implies that gut microbial metabolites may play a supportive role in the therapeutic efficacy of Acu treatment (57–59). This suggests a mechanistic link whereby microbial metabolites modulate host neurophysiology, potentially enhancing Acu-induced neuromodulation via the gut-brain-skin axis.

Stratifying patients based on microbiome profiles has been shown to be a powerful approach for understanding variable therapeutic outcomes across diseases, including cancer and inflammatory disorders (60, 61). While previous studies have primarily focused on differences between AD patients and healthy controls (62, 63), our study uniquely stratifies AD patients based on their response to Acu and reveals microbial signatures directly associated with therapeutic responsiveness. The experiment with R-enriched microbial signatures demonstrated that the therapeutic effect of Acu is mediated, at least in part, through the gut microbiota. Identifying gut microbiome biomarkers enables the development of patient-specific therapeutic strategies, which improve treatment outcomes in individuals with AD. This highlights the potential of applying Acu treatment in a personalized manner, especially for patients requiring long-term AD management.

Several limitations of this study should be acknowledged. First, while our baseline-FMT design causally demonstrates that the gut microbial community is sufficient to confer differential acupuncture responsiveness, we did not include mid-treatment microbial sampling or an FMT arm using post-treatment donor samples. We therefore cannot exclude the possibility that acupuncture additionally induces subtle longitudinal remodeling of the microbiota in responders that contributes to sustained efficacy. Future longitudinal studies incorporating densely sampled time points and post-treatment FMT arms will be needed to resolve the temporal dynamics of the acupuncture-microbiome interaction. Second, the exclusive use of male mice in our AD model may limit the translational applicability of our findings, as AD pathology and immune responses can differ substantially between sexes; subsequent work should incorporate both sexes to capture potential sex-dependent effects. Finally, our focus on a single disease constrains the generalizability of the identified microbial signatures. Diverse, multi-center cohorts across different disease contexts will be required to determine whether microbiome biomarkers associated with acupuncture responsiveness in AD are broadly applicable to other neuroimmune-related conditions.

## Data Availability

All data and code will be shared upon request via the corresponding author. The 16S rRNA and WMS raw sequence files are deposited in the National Center for Biotechnology Information (NCBI) Sequence Read Archive (SRA) under SRP588096 of BioProject ID PRJNA1268706.

## SUPPLEMENTAL MATERIAL

Supplemental Material spectrum.03912-25-s0001.pdf Fig. S1 to S5; Tables S1 and S2.

ASM does not own the copyrights to Supplemental Material that may be linked to, or accessed through, an article. The authors have granted ASM a non-exclusive, world-wide license to publish the Supplemental Material files. Please contact the corresponding author directly for reuse.
