# Supplementary material for: Gut microbiota contributes to the therapeutic effect of acupuncture in atopic dermatitis
Source: Microbiol Spectr. 2026 Aug 3;14(9):e03912-25. doi: 10.1128/spectrum.03912-25 (PMC13532318; doi:10.1128/spectrum.03912-25)
Supplement: Supplementary file 1 [file spectrum.03912-25-s0001.pdf]

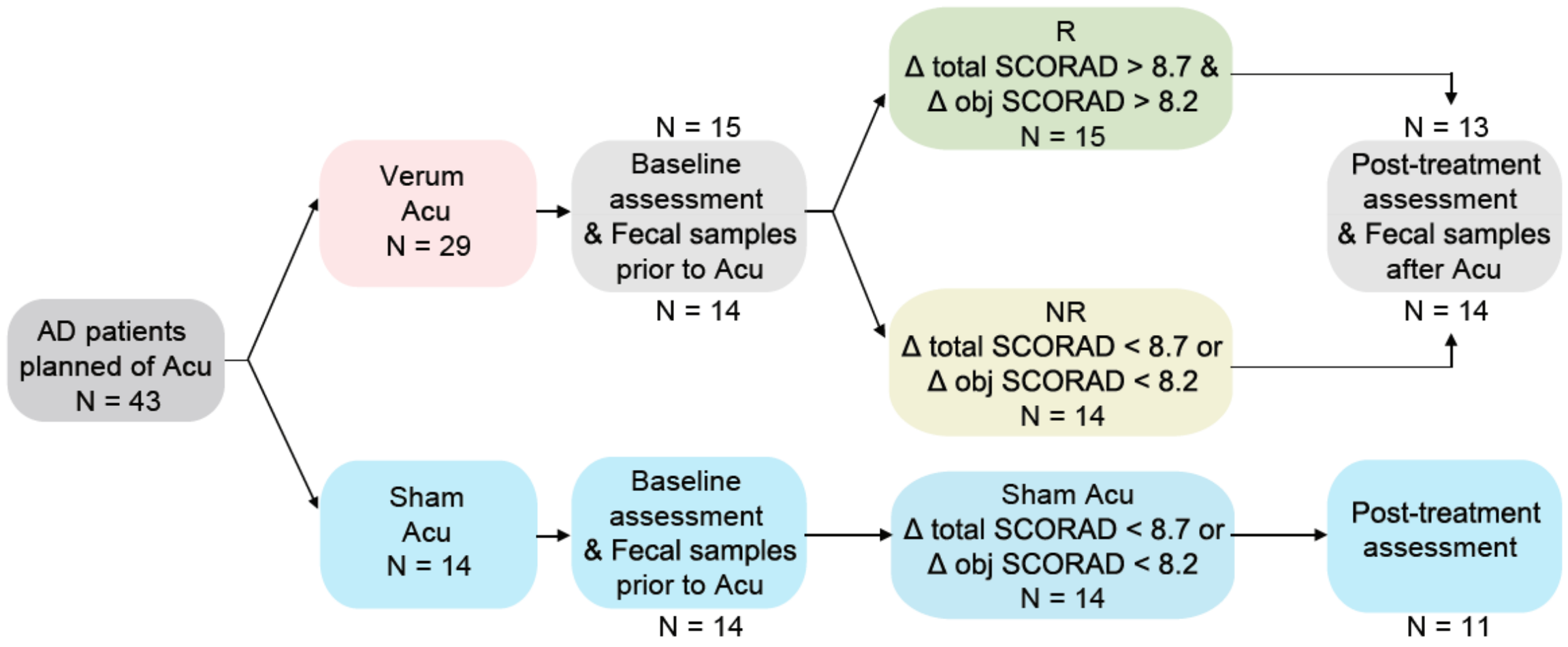


**Figure S1. Clinical grouping and outcome tracking of AD patients undergoing acupuncture treatment**

Flow diagram showing the clinical course and grouping of AD patients planned for Acu treatment. AD patients were divided into verum Acu and sham Acu groups. Among verum Acu-treated AD patients, those with a change in total SCORAD > 8.7 and a change in objective SCORAD > 8.2 were classified as R, while others were categorized as NR. Baseline fecal samples and baseline assessments were obtained prior to Acu treatment, and post-fecal sample and post-treatment assessments were conducted after Acu treatment.

Acu, acupuncture; SCORAD, scoring of atopic dermatitis; R, responder; NR, non-responder

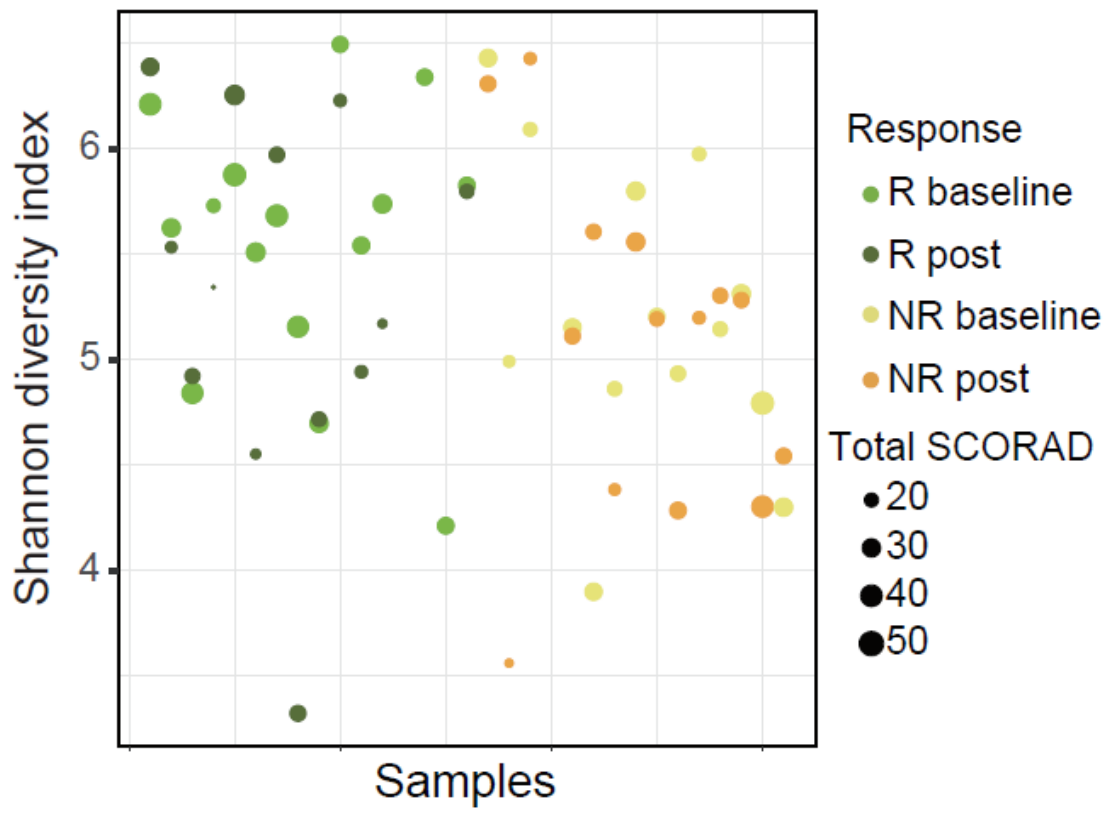


**Figure S2. Alpha and beta diversity of gut microbiota in AD patients before and after acupuncture treatment**

Scatter plot illustrating the relationship between shannon diversity index and total SCORAD across all samples. Each dot represents a sample, with circle size indicating SCORAD severity. 'Baseline' matched samples before Acu treatment (pre-samples).
SCORAD, scoring of atopic dermatitis

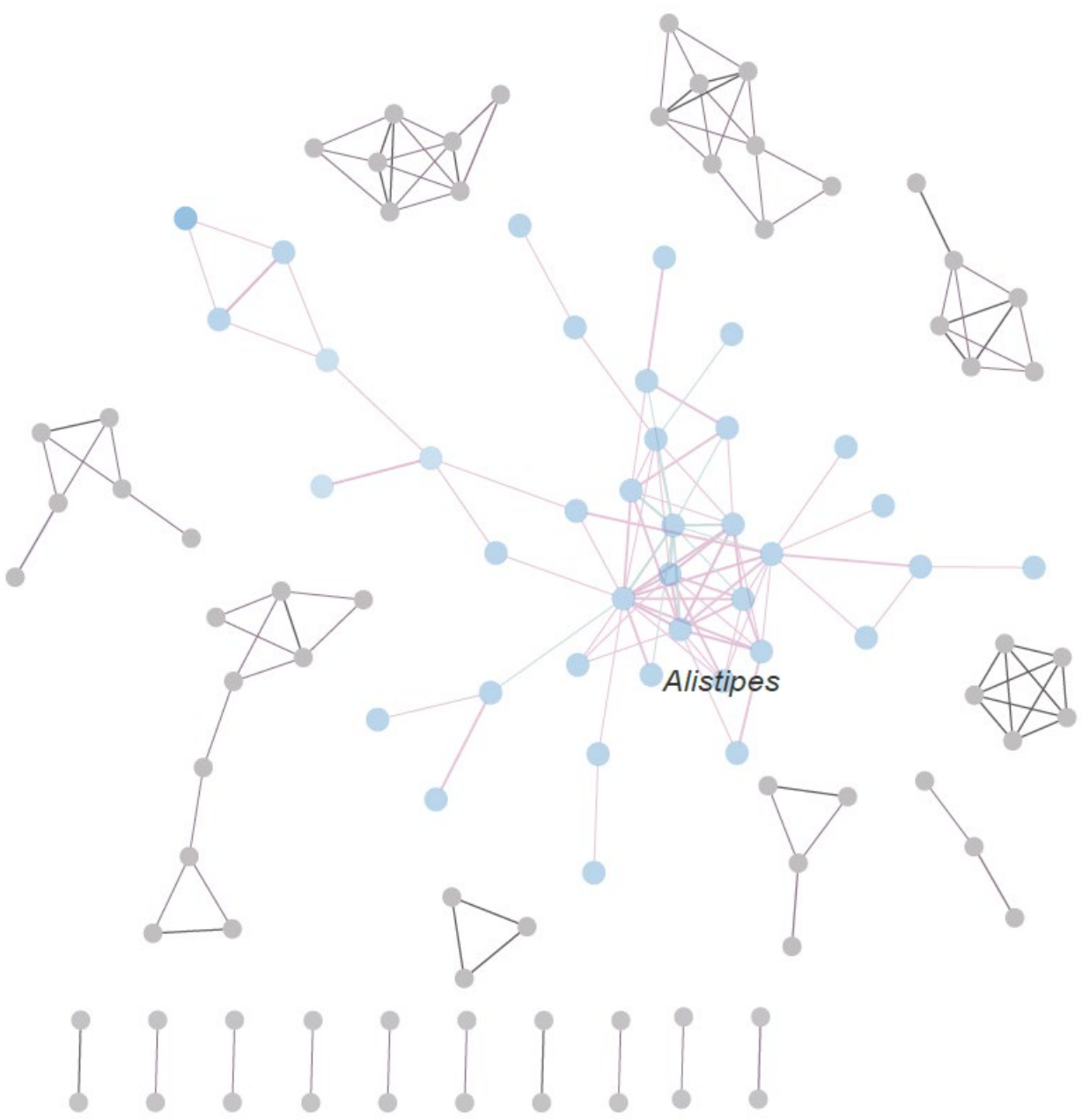


**Figure S3. Enriched microbial taxa in responders form a core network cluster associated with treatment responsiveness.**

Network of 261 bacterial genera based on Spearman correlation in AD patients before Acu treatment. Colored-blue nodes represent core-nodes.

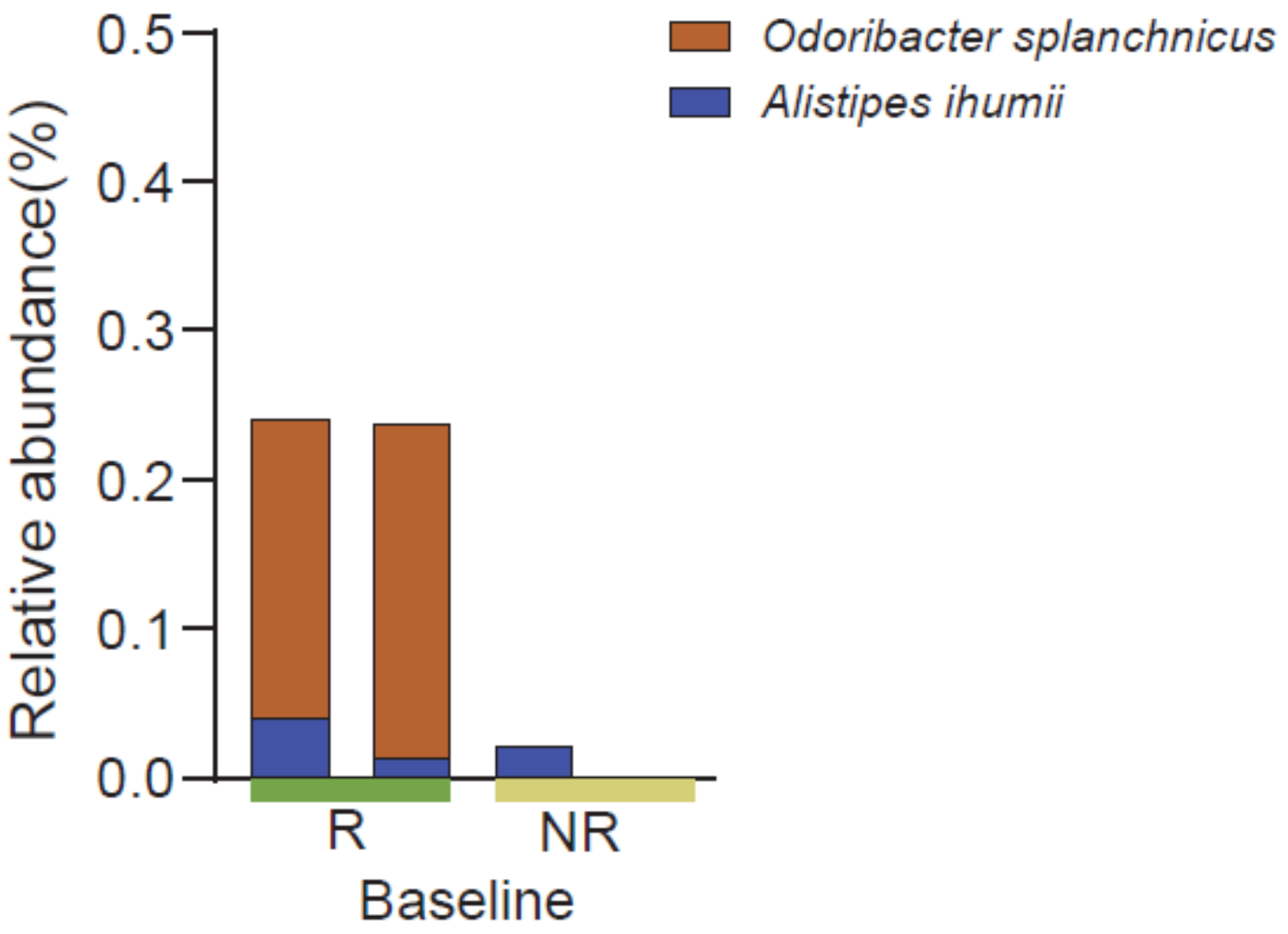


**Figure S4. Comparison of relative abundance (%) in 4 fecal samples from fecal microbiota transplantation experiment using 16S rRNA amplicon sequencing data**

Comparison of microbial genus- and species-taxa enriched in the R groups in 4 fecal samples from the FMT experiment. 'Baseline' matched samples before Acu treatment (pre-samples). FMT, fecal microbiota transplantation.

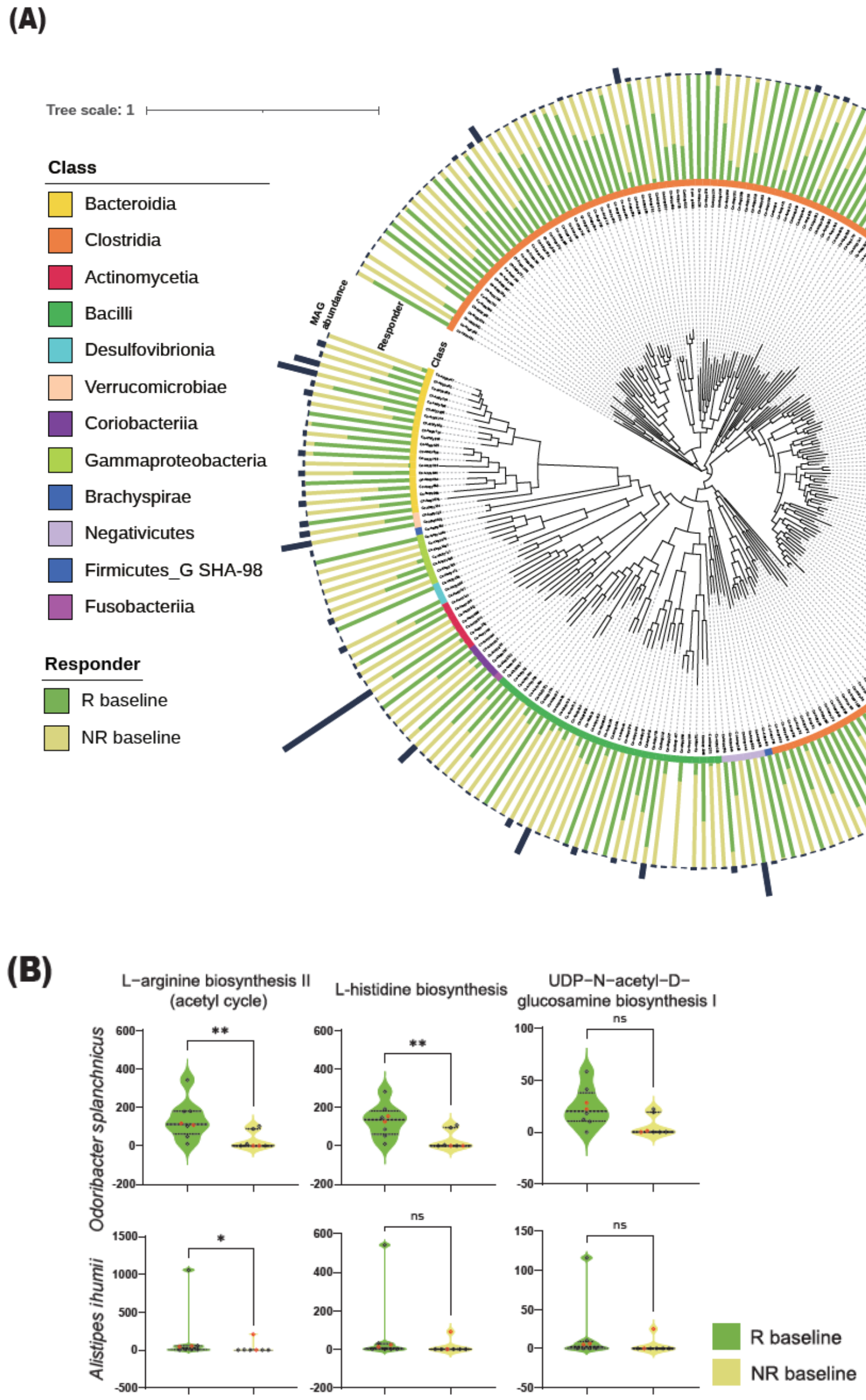


**Figure S5. Enrichment of several microbia l metabolic pathways in the responder group based on metagenom-assembled genome's gene count data and their association with biomarker taxa**

(A) Maximum likelihood phylogenetic tree of all bacterial species-level metagenome-assembled genomes (MAGs) constructed from fecal samples before Acu treatment. The middle ring indicates the responder group origin (green: R pre; yellow: NR pre), allowing visualization of MAG distribution across response status. The outermost ring represents abundance of each MAG across all samples, with bar height corresponding to abundance levels.

(B) Violin plots illustrating several microbial metabolic pathways across R-baseline and NR-baseline groups. Each panel displays pathway-specific gene abundances calculated from MAG of WMS data. Red dots indicate fecal samples from FMT experiment.

WMS, whole metagenome sequencing.

**Table S1. Clinical characteristics and indices of R and NR groups treated with verum Acu, whose fecal samples were subjected to WMS.**

| | | R (N=6) | NR (N=5) | *p-value* |
|---|---|---|---|---|
| Age | | 23.5 ± 2.8 | 23.0 ± 1.9 | 0.743 |
| Sex | | | | 0.329 |
| | -Male | 7 (46.7%) | 10 (71.4%) | |
| Height | | 170.0 ± 8.6 | 167.4 ± 8.6 | 0.628 |
| Weight | | 65.6 ± 12.5 | 62.0 ± 11.0 | 0.626 |
| BMI | | 22.6 ± 3.2 | 22.0 ± 2.2 | 0.735 |
| BDI | | 25.5 ± 2.9 | 25.2 ± 4.1 | 0.891 |
| GSRS | | | | |
| | - Baseline | 31.5 ± 10.9 | 25.8 ± 5.4 | 0.315 |
| | - Post | 17.5 ± 11.0 | 22.6 ± 4.3 | 0.359 |
| VAS itching | | | | |
| | - Baseline | 5.6 ± 1.1 | 5.3 ± 1.7 | 0.792 |
| | - Post | 2.6 ± 1.8 | 4.4 ± 2.0 | 0.142 |
| VAS insomnia | | | | |
| | - Pre | 4.1 ± 1.4 | 2.5 ± 1.2 | 0.075 |
| | - Post | 0.6 ± 0.7 | 2.3 ± 1.5 | 0.038 |
| SCORAD total | | | | |
| | - Baseline | 41.3 ± 5.6 | 23.7 ± 4.0 | 0 |
| | - Post | 21.5 ± 6.2 | 22.6 ± 6.3 | 0.773 |
| SCORAD object | | | | |
| | - Baseline | 31.8 ± 6.7 | 15.9 ± 3.0 | 0.001 |
| | - Post | 18.4 ± 7.4 | 15.9 ± 5.8 | 0.562 |
| EASI | | | | |
| | - Baseline | 6.0 ± 3.2 | 3.4 ± 1.5 | 0.121 |
| | - Post | 3.0 ± 2.6 | 2.9 ± 1.3 | 0.907 |
| TARC (pg/mL) | | | | |
| | - Baseline | 406.6 ± 258.8 | 413.2 ± 31.0 | 0.953 |
| | - Post | 206.6 ± 91.0 | 257.0 ± 159.6 | 0.526 |
| Autotaxin (pg/mL) | | | | |
| | - Baseline | 256.4 ± 53.6 | 245.5 ± 25.1 | 0.686 |
| | - Post | 210.8 ± 19.9 | 173.7 ± 102.1 | 0.467 |
| DLQI | | | | |
| | - Baseline | 9.5 ± 4.5 | 8.4 ± 4.6 | 0.696 |
| | - Post | 4.3 ± 2.1 | 4.2 ± 2.3 | 0.921 |
| Credibility test | | | | |
| | - Baseline | 19.7 ± 2.9 | 18.4 ± 3.6 | 0.539 |
| | - Post | 20.5 ± 1.9 | 15.6 ± 6.3 | 0.162 |

BMI; body mass index
BDI; beck depression inventory
GSRS; gastrointestinal symptom rating scale
VAS; visual analogue scale score
SCRAD; scoring atopic dermatitis
EASI; eczema area and severity index
TARC; thymus and activation-regulated chemokine
DLQI; the dermatology life quality index

WMS; whole metagenome sequencing

**Table S2. Clinical characteristics and indices of AD patients treated with verum and sham Acu.**

| | | Sham Acu (N=14) | Verum Acu (N=29) | *p-value* |
|---|---|---|---|---|
| Age | | 24.0 ± 4.2 | 24.8 ± 5.8 | 0.626 |
| Sex | | | | 0.403 |
| | -Male | 7 (43.8%) | 19 (61.3%) | |
| Height | | 166.6 ± 7.2 | 168.8 ± 9.1 | 0.402 |
| Weight | | 66.8 ± 13.9 | 67.1 ± 12.6 | 0.953 |
| BMI | | 23.9 ± 3.9 | 23.4 ± 2.9 | 0.594 |
| BDI | | 27.5 ± 5.7 | 26.2 ± 4.3 | 0.384 |
| GSRS | | | | |
| | - Baseline | 29.1 ± 9.6 | 28.2 ± 8.1 | 0.754 |
| | - Post | 23.9 ± 11.3 | 22.0 ± 10.1 | 0.62 |
| VAS itching | | | | |
| | - Baseline | 5.7 ± 2.0 | 5.8 ± 1.6 | 0.908 |
| | - Post | 3.6 ± 1.8 | 3.8 ± 2.1 | 0.819 |
| VAS insomnia | | | | |
| | - Baseline | 3.0 ± 2.5 | 3.4 ± 1.8 | 0.47 |
| | - Post | 2.0 ± 2.1 | 1.9 ± 1.9 | 0.872 |
| SCORAD total | | | | |
| | - Baseline | 31.1 ± 10.2 | 33.8 ± 8.9 | 0.353 |
| | - Post | 31.7 ± 12.7 | 24.8 ± 7.9 | 0.051 |
| SCORAD object | | | | |
| | - Baseline | 22.5 ± 8.7 | 24.7 ± 8.3 | 0.406 |
| | - Post | 26.0 ± 10.4 | 19.1 ± 7.0 | 0.022 |
| EASI | | | | |
| | - Baseline | 4.7 ± 4.1 | 5.8 ± 4.1 | 0.386 |
| | - Post | 6.8 ± 6.0 | 4.3 ± 4.4 | 0.165 |
| TARC (pg/mL) | | | | |
| | - Baseline | 251.7 ± 140.9 | 348.2 ± 154.8 | 0.043 |
| | - Post | 159.0 ± 84.8 | 198.1 ± 116.4 | 0.32 |
| Autotaxin (pg/mL) | | | | |
| | - Baseline | 240.2 ± 76.4 | 247.6 ± 38.7 | 0.719 |
| | - Post | 210.3 ± 83.0 | 206.0 ± 58.6 | 0.857 |
| DLQI | | | | |
| | - Baseline | 7.0 ± 5.0 | 9.1 ± 4.6 | 0.158 |
| | - Post | 6.6 ± 6.2 | 5.1 ± 3.2 | 0.451 |
| Credibility test | | | | |
| | - Baseline | 17.5 ± 1.8 | 17.7 ± 3.3 | 0.812 |
| | - Post | 17.2 ± 2.6 | 17.7 ± 4.1 | 0.678 |

BMI; body mass index
BDI; beck depression inventory
GSRS; gastrointestinal symptom rating scale
VAS; visual analogue scale score
SCRAD; scoring atopic dermatitis
EASI; eczema area and severity index
TARC; thymus and activation-regulated chemokine
DLQI; the dermatology life quality index

AD; atopic dermatitis, Acu; acupuncture
